# The effect of shared decision-making for critically ill patients: a systematic review and meta-analysis

**DOI:** 10.3389/fmed.2026.1726976

**Published:** 2026-01-30

**Authors:** Yangyang Wang, Jiaqi Li, Na Yin, Baolin Huang, Kaibo Shen, Sheng He, Minfei Yang, Ju Zhang

**Affiliations:** 1 Department of Public Health and Nursing, Hangzhou Normal University, Zhejiang, China; 2 Department of Nursing, The Second Affiliated Hospital of Zhejiang University School of Medicine, Zhejiang, China

**Keywords:** critical, critical ill patients, effect, shared decision-making, systematic review and meta-analysis

## Abstract

**Objective:**

This systematic review and meta-analysis assessed the impact of shared decision-making on critically ill patients, focusing on outcomes such as mortality, intensive care unit (ICU) and hospital length of stay (LOS), and mental health symptoms in patients and their surrogates.

**Methods:**

Following PRISMA guidelines, we searched PubMed, EMBASE, Web of Science, and Cochrane databases through March 2025 for randomized controlled trials (RCTs) assessing shared decision-making interventions in critically ill patients or surrogates. Risk of bias was assessed using the Cochrane tool, and data synthesis employed fixed or random-effects models based on heterogeneity.

**Results:**

Fifteen RCTs (2003–2025) involving 3,678 ICU patients and 2,777 surrogates were analyzed. Shared decision-making showed no significant association with all-cause mortality [risk ratio (RR) 1.05, 95% CI = 0.97–1.15]. Data analysis shows that the ICU LOS for deceased patients have shortened [standardized mean difference (SMD) = −0.15, 95% CI = −0.27 to −0.02, *p* = 0.02], but no effect on overall ICU LOS (SMD = 0.02, 95% CI = −0.06 to 0.10, *p* = 0.64) or hospital LOS (SMD = 0.02, 95% CI = −0.06 to 0.10, *p* = 0.64). Shared decision-making demonstrated no benefits for surrogate mental health outcomes, including depression (SMD = −0.04, 95% CI = −0.18 to 0.10, *p* = 0.57), anxiety (SMD = 0.06, 95% CI = −0.22 to 0.34, *p* = 0.69), or PTSD symptoms (SMD = −0.08, 95% CI = −0.37 to 0.21, *p* = 0.57). Decision-making quality (SMD = 0.02, 95% CI = −0.15 to 0.19, *p* = 0.81) and communication quality (SMD = 0.09, 95% CI = −0.09 to 0.27, *p* = 0.33) remained unchanged.

**Conclusion:**

Shared decision-making may reduce ICU LOS for critically ill patients who ultimately die, without influencing mortality or overall hospitalization duration. Culturally tailored shared decision-making interventions are needed to address the heterogeneous needs of patients and surrogates across diverse populations.

## 1 Background

Shared decision-making, a collaborative process integrating clinical expertise with patient and surrogate values, seeks to align treatment choices with patient preferences while reducing decisional conflict (1). In critical care settings, where patients often lack decision-making capacity, physicians rely on proxies (e.g., family members) to facilitate shared decision-making (2). However, time constraints, prognostic uncertainty, and surrogate emotional burdens frequently compromise shared decision-making effectiveness in intensive care units (ICUs) (3). Institutional barriers, including clinician time limitations, inadequate training, and suboptimal communication environments, further hinder implementation (4).

Shared decision-making interventions, ranging from structured family conferences and decision aids to ethics consultations and palliative care guidance (5, 6)—aim to reconcile treatment plans with patient values and alleviate surrogate distress. Despite improving transparency, shared decision-making often struggles to address the urgency and complexity of ICU decisions. For instance, surrogate decision-makers for patients with severe acute brain injury requiring prolonged mechanical ventilation report persistent anxiety and depression (7), while those managing chronic critical illness face elevated post-traumatic stress disorder (PTSD) risks (8). Although some evidence suggests shared decision-making may reduce ICU length of stay (LOS), findings remain inconsistent.

Prior meta-analyses report conflicting outcomes: ICU-focused shared decision-making interventions may shorten ICU stays without affecting mortality (9), whereas others propose mortality and hospital LOS reductions (10). Paradoxically, decision aids might prolong hospitalization despite enhancing surrogate knowledge (11). These discrepancies likely stem from heterogeneous study designs, populations, and outcome measures. To resolve this uncertainty, we systematically analyzed randomized controlled trials (RCTs) evaluating shared decision-making’s impact on mortality, psychosocial outcomes, and healthcare utilization in critically ill patients and their surrogates.

## 2 Methods

This study followed the Cochrane Handbook for Systematic Reviews of Interventions (12) and was reported in accordance with the PRISMA guidelines (13). The protocol is registered with the International Prospective Register of Systematic Reviews (CRD420251013215).^1^

### 2.1 Data sources and search strategy

A comprehensive search was conducted in March 2025 across PubMed, EMBASE, Web of Science, and Cochrane databases, from inception to March 2025. Search terms included Medical Subject Headings (MeSH) and key terms such as “critical illness,” “shared decision-making,” “decision aids,” and “family.” The full search strategy is detailed in Supplementary Tables S1–S4. We also manually searched reference lists of relevant studies and reviews (14–16).

### 2.2 Inclusion and exclusion criteria

Eligible studies were screened based on the following criteria:

*Population:* Critically ill patients (≥18 years) or their surrogates (e.g., family members).

*Intervention:* Any form of shared decision-making between patients, doctors, and family members.

*Control:* Routine care or no intervention.

*Outcomes:* All-cause mortality, ICU length of stay (LOS), hospital LOS, depression, anxiety, PTSD symptoms, decision-making quality and communication quality.

*Study Design:* Randomized controlled trials (RCTs) published in English, with no date restrictions.

Studies were excluded if they: (1) Interventions targeted only at healthcare professionals (doctors or nurses); (2) Qualitative studies, protocols, conference abstracts, case reports, letters, reviews; (3) Cross-over controlled RCTs; (4) Absence of the outcomes of interest; (5) Lack of the full text or the relevant complete study.

### 2.3 Study selection

Two reviewers (WYY and LJQ) independently screened all titles and abstracts, resolving discrepancies through consensus with a third reviewer. Only studies meeting all inclusion criteria proceeded to full-text review and data extraction.

### 2.4 Data extraction

Two reviewers (WYY and LJQ) independently extracted data using standardized forms, with a third reviewer (ZJ) verifying accuracy. Any discrepancies were resolved through discussion. Data collected included study characteristics (author, year, country, design, sample size), participant characteristics, intervention details, and outcome data.

### 2.5 Intervention categorization and rationale for synthesis

We recognized that the shared decision-making intervention measures included in the studies were heterogeneous in form and implementation. To address this issue and provide a basis for data pooling, we classified the intervention measures into four categories based on their core components.

#### 2.5.1 Structured communication or family meeting

Interventions involving dedicated, protocol-driven meetings or communication support led by trained personnel (for example, nurses, palliative care specialists, ethics consultants) to facilitate family-clinician dialogue.

#### 2.5.2 Decision tools

Interventions employing paper-based or electronic tools (for example, pamphlets, worksheets, web-based platforms) designed to provide prognostic information, clarify patient values, and prepare surrogates for decision-making.

#### 2.5.3 Integrated interventions

Interventions combining elements of structured communication with additional supportive components, such as family navigation, spiritual care, or specific clinical management guidance.

#### 2.5.4 Palliative care consultations

Formal consultations conducted by an institutional ethics committee or a specialized palliative care team to address treatment conflicts or goals-of-care discussions.

### 2.6 Risk of bias

The Cochrane Risk of Bias tool (17) was used by two reviewers to assess the methodological quality of each study. The assessment covered random sequence generation, allocation concealment, blinding, outcome assessment, incomplete data, selective reporting, and other potential biases. Discrepancies were resolved by a third reviewer.

### 2.7 Data synthesis and analysis

Meta-analysis was performed using Review Manager 5.4. Binary outcomes were expressed as Risk Ratios (RR) with 95% confidence intervals (CI), while continuous outcomes were reported as mean difference (MD) or standardized mean difference (SMD) with 95% CI. For continuous outcomes, if different studies used the same unit of measurement but different scales, or different units of measurement, SMD was selected as the effect size indicator; SMD was calculated based on the mean, standard deviation, and sample size of each study through the built-in algorithm of Review Manager 5.4 software, reflecting the standardized difference in outcome indicators between the intervention group and the control group. For studies reporting only median and interquartile range, raw data were sought from authors, and where unavailable, McGrath’s method was used to convert medians to means and standard deviations (18, 19). Handling of studies with multiple time points: If the included studies reported the same outcome indicator at multiple time points, the time point consistent with the definition of the study’s primary outcome, was prioritized for data extraction and pooling; if the study did not clearly define the primary time point, the time point closest to the end of the intervention was selected. All 15 randomized controlled trials included in this study were two-arm trials, and no multi-arm trials were involved, so no data processing related to multi-arm trials was required. Fixed-effect models were used when I^2^ < 50% and *p* ≥ 0.1, indicating low heterogeneity. For significant heterogeneity (I^2^ > 50% or *p* < 0.1), random-effects models were applied, and subgroup analyses were conducted to explore sources of heterogeneity. Subgroup variables included all-cause mortality, surrogate symptoms (depression, anxiety, PTSD), ICU and hospital LOS, decision-making and communication quality, and mechanical ventilation duration. A *p*-value < 0.05 was considered statistically significant in subgroup analyses. A sensitivity analysis was performed to assess result robustness by sequentially excluding studies, and publication bias was evaluated using Egger’s test, with a *p* > 0.05 considered as no significant publication bias. The results of Egger’s test for each outcome are reported in the corresponding result sections (Supplementary Figures S2, S7).

### 2.8 Assessment of the quality of evidence

We used the Grading of Recommendations, Assessment, Development and Evaluation (GRADE) approach to assess the quality of evidence of the included studies (20) (Supplementary Table S5).

## 3 Results

### 3.1 Literature selection process

**Figure 1:**
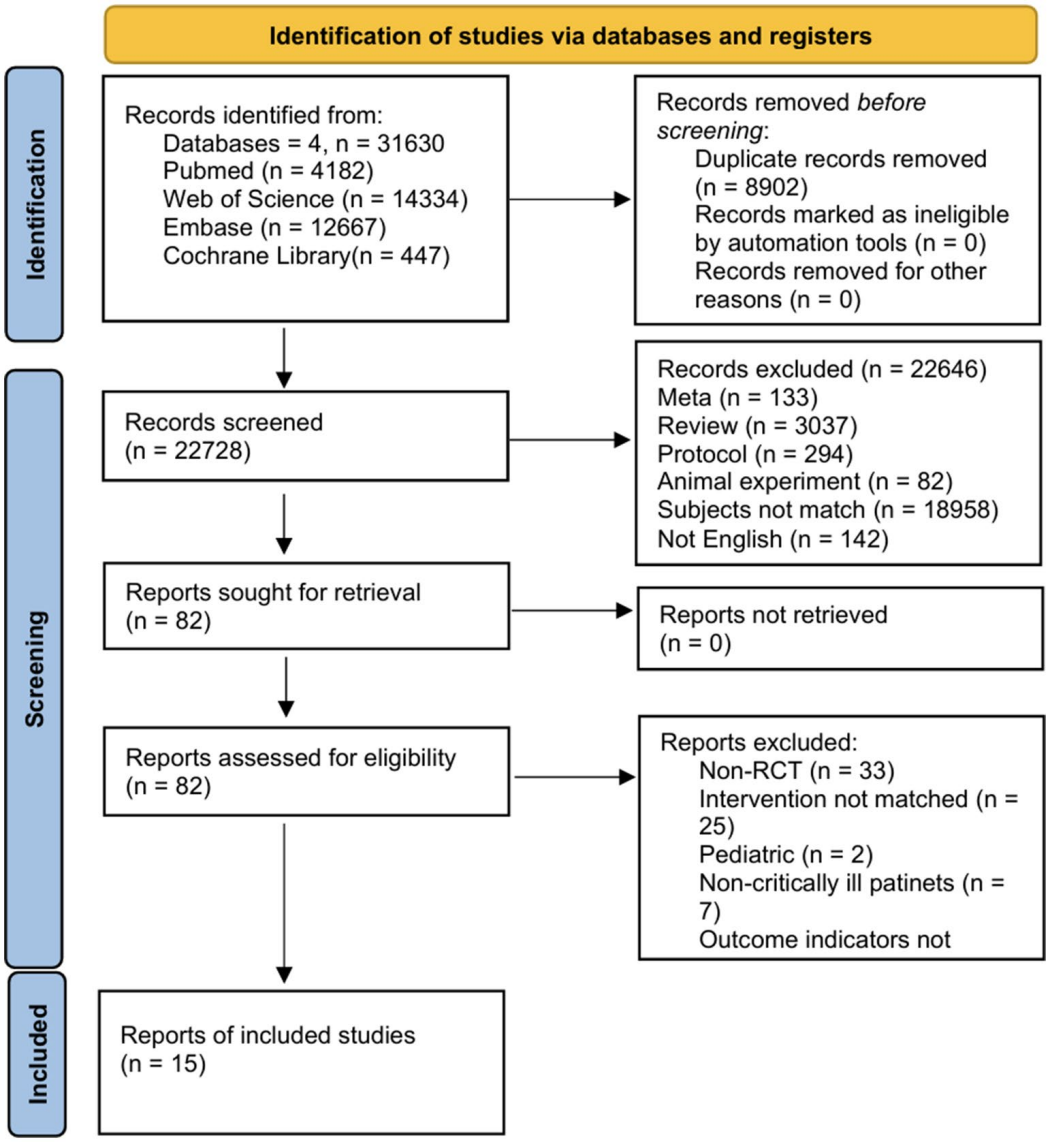
Flow chat for selecting the articles in this systematic review.

The initial search identified 31,630 records, supplemented by 3 additional articles from manual searching. After screening, 15 RCTs were included in the final analysis (Figure 1).

### 3.2 Study characteristics

Table 1 presents the characteristics of the 15 RCTs. These studies, published between 2003 and 2025, involved 3,678 ICU patients and 2,777 surrogates. Eleven studies (14, 15, 21–29) were conducted in the U.S., two (30, 31) in Australia, and two (16, 32) in France. The studies included both single-center (6) and multi-center (9) settings, with patient ages ranging from 34.9 to 97 years and surrogates’ ages from 34 to 74.2 years. Various outcomes were reported, including mortality, surrogate depression, anxiety, PTSD, ICU and hospital length of stay, quality of decision-making, and mechanical ventilation duration.

**Table 1:** Basic characteristics of the included studies.

Author, year, country	Sites	Participants	Intervention	Types of interventions	No. of participants (experimental/control)		Age (years, experimental/control, mean ± SD)		Outcome and outcome measure
					Patients (Male/Female)	Shared decision-makings (Male/Female)	Patients	Shared decision-makings	
Cox et al. (22), US	Multicenter RCT of 6 ICUs	Critically ill older adult patients and their family members with elevated palliative care needs	An automated electronic health record–integrated, mobile application–based communication platform	Decision tools	76(42/34)/75(44/31)	76(22/53)/75(18/57)	69.2 ± 9.8/70.5 ± 9.6	57.3 ± 12.9/57.4 ± 13.0	Depression, anxiety, surrogates’ symptoms of posttraumatic stress disorder, ICU LOS (all patients), hospital LOS, overall QOC
Butler et al. (23), US	Multicenter RCT of 6 ICUs	Patients ≥21 years old, with >40% risk of in-hospital death or long-term impairment, dependent on >2 ADLs, and their surrogates	Four Supports intervention adds: emotional support; communication support; decisional support	Integrated interventions	146(75/71)/145(88/57)	233(55/168)/209(63/146)	61.0 ± 17.1/62.2 ± 15.6	51.1 ± 15.0/53.4 ± 15.2	Hospital mortality, surrogates’ symptoms of posttraumatic stress disorder, ICU LOS (all patients), ICU LOS (patients who died), hospital LOS, quality of decision-making surrogate
Marshall et al. (31), Australia	Multicenter RCT of 9 ICUs	ICU patients who were nutritionally high-risk and/or those at risk of dying in the ICU or during subsequent hospitalization and their adult family members	Nutrition intervention and decision support intervention	Integrated interventions	40(25/15)/44(31/13)	40(12/28)/44(11/33)	73.0 ± 8.0/71.5 ± 8.2	56.4 ± 16.2/58.1 ± 16.1	Hospital mortality, ICU mortality, hospital LOS
Muehlschlegel et al. (24), US	Multicenter RCT of 2 ICUs	Critically ill patients with severe acute brain injury and their surrogates	Provided a technical orientation of the decision-aid to the intervention group surrogates without any mention of medical information and reminded them to complete the worksheet	Decision tools	20(11/9)/21(15/6)	33(5/27)/33(11/22)	58 ± 19/64 ± 21	51 ± 17/57 ± 15	Hospital mortality, depression, anxiety, surrogates’ symptoms of posttraumatic stress disorder, ICU LOS (all patients), hospital LOS, quality of decision-making surrogate, overall QOC, length of mechanical ventilation
Suen et al. (25), US	Single center RCT of 2 ICUs	Critical patients in ICU and their surrogates	Surrogates complete Family Support Tool before meetings, provide summary to ICU team, and schedule family meetings	Structured communication or family meeting	25(13/12)/25(11/14)	23(6/17)/25(12/13)	65.6 ± 17.7/69 ± 13.2	58.65 ± 12.62/54.28 ± 13.15	Overall QOC
Robin et al. (32), France	Single center RCT of 3 ICUs	Adult patients for whom a decision to withhold and withdraw life-sustaining therapies in the ICU	Information pamphlet	Decision tools	45(28/17)/45(29/16)	45(20/25)/45(14/31)	69 (62–77)/71 (59–80)	54 (47–65)/54 (47–66)	Depression, anxiety, surrogates’ symptoms of posttraumatic stress disorder
Alghanim et al. (26), US	Single center RCT of 2 ICUs	Critical patients in MICU or CICU and their surrogates	Chaplain patient navigator served as a health liaison for patients and their families, elicited their values and preferences, and organized admission, follow-up, and *ad hoc* family meetings	Integrated interventions	601(334/266)/573(287/286)	Not mentioned	63(53–76)/62(53–75)	Not mentioned	ICU LOS (all patients), ICU LOS (patients who died), hospital LOS
Cox et al. (27), US	Multicenter RCT of 5 hospital	Adult patients receiving prolonged mechanical ventilation and their surrogates	Web-based decision aid provided prognostic estimates, treatment options, and clarified patient values for family meetings.	Decision tools	138(88/50)/139(89/50)	137(41/96)/138(33/105)	52.9 ± 17.9/54.0 ± 16.6	49.9 ± 13.5/52.6 ± 11.6	Hospital mortality, depression, anxiety, surrogates’ symptoms of posttraumatic stress disorder, ICU LOS (all patients), hospital LOS, quality of decision-making surrogate, overall QOC, length of mechanical ventilation
Torke et al. (28), US	Single center RCT	Sedated or comatose ICU patients	Dedicated trained nurse acting as family navigator	Structured communication or family meeting	13(4/9)/13(7/6)	13(9/4)/13(3/10)	53.27 (14.18)/57.42 (11.03)	50.93 (12.01)/46.16 (17.36)	Depression, anxiety, quality of decision-making surrogate
Curtis et al. (29), US	Multicenter RCT of 2 ICUs	ICU patients and family members	A communication facilitator enhances ICU communication self-efficacy for families and clinicians through interviews, meetings, and follow-up	Structured communication or family meeting	82(55/27)/86(53/33)	131(38/93)/137(41/96)	52.1 ± 17.2/55.3 ± 18.8	49.5 ± 12.0/52.4 ± 14.2	Hospital mortality, ICU LOS (all patients), ICU LOS (patients who died)
Carson et al. (21), US	Multicenter RCT of 4 ICUs	Adult patients (≥21 years) requiring 7 days of mechanical ventilation, with their family surrogates enrolled in the study	At least 2 structured family meetings led by palliative care specialists and provision of an informational brochure	Integrated interventions	130(64/66)/120(55/65)	184(56/128)/181(50/131)	58 (55.2–60.8)/57 (54.0–59.7)	51 (48.8–52.8)/51 (48.6–52.7)	Hospital mortality, depression, anxiety, surrogates’ symptoms of posttraumatic stress disorder, ICU LOS (all patients), ICU LOS (patients who died), hospital LOS, length of mechanical ventilation
Cheung et al. (30), Australia	Single center RCT	Patients with terminal or preterminal conditions, where escalating or continuing treatment is unlikely to improve their clinical condition	A consultation and subsequent management by a palliative care team	Palliative care consultations	10(5/5)/10(3/7)	共9	72(20)/83(14)	Total 9	Hospital mortality, ICU mortality, ICU LOS (all patients), ICU LOS (patients who died)
Andereck et al. (15), US	Single center RCT of medical/surgical ICU	Patients with ICU lengths of stay of 5 days or greater	Ethics: Proactive ethics intervention involves a trained bioethicist in the care of all ICU patients	Palliative care consultations	174(83/91)/210(98/112)	146/173	60 ± 15.8/61 ± 16.2	Not reported	Hospital mortality
Lautrette et al. (16), France	Multicenter RCT of 22 ICUs	Patient would die within a few days	A proactive end-of-life conference and a brochure	Integrated interventions	63(33/30)/63(37/26)	57(17/40)/52(12/40)	74(56–80)/68(56–76)	54(47–58)/54(46–64)	Hospital mortality, surrogates’ symptoms of posttraumatic stress disorder, ICU LOS (all patients), ICU LOS (patients who died)
Schneiderman et al. (14), US	Multicenter RCT of 7 hospitals	Patient in whom treatment conflicts were identified	Ethics consultation offered	Palliative care consultations	276(145/131)/270(148/122)	262/263	67.5 ± 17.2/67.5 ± 17.4	Not reported	Hospital mortality, ICU LOS (patients who died), hospital LOS, length of mechanical ventilation

Three studies (25, 28, 29) employed structured communication or family meetings as the intervention methods, four studies (22, 24, 27, 32) employed decision Tools, five studies (16, 21, 23, 26, 31) employed Integrated Interventions, three studies (14, 15, 30) employed palliative care consultations.

### 3.3 Risk of bias

**Figure 2:**
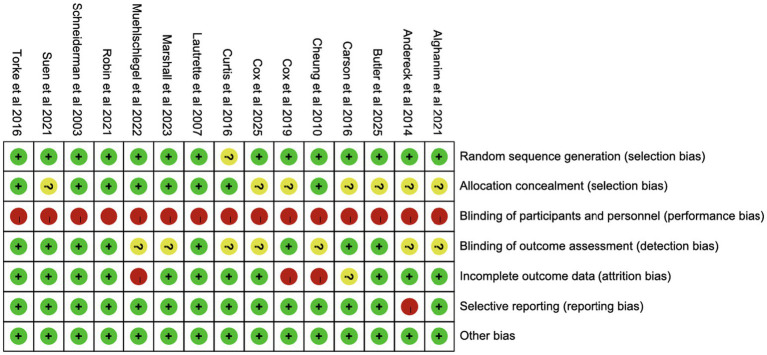
Risk of summary.

**Figure 3:**
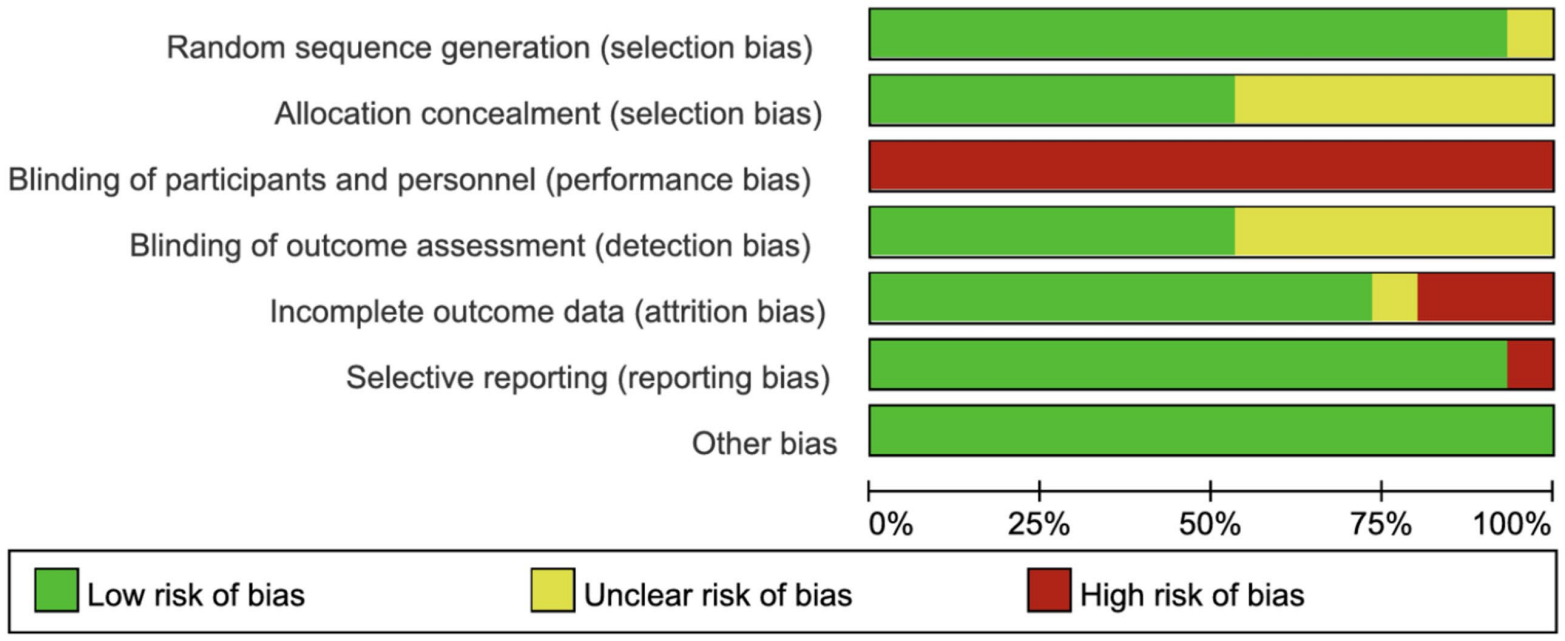
Risk of graph.

Except for one study lacking details on random sequence generation, most studies reported adequate randomization. Eight studies exhibited low risk of selection bias, while seven did not specify allocation concealment. None of the trials blinded participants or staff, but eight blinded outcome assessments. Three studies had high follow-up loss, and one study did not report follow-up rates. One study lacked information on public registration or protocol availability (Figures 2, 3).

### 3.4 Meta-analysis

#### 3.4.1 All-cause mortality in different environments

**Figure 4:**
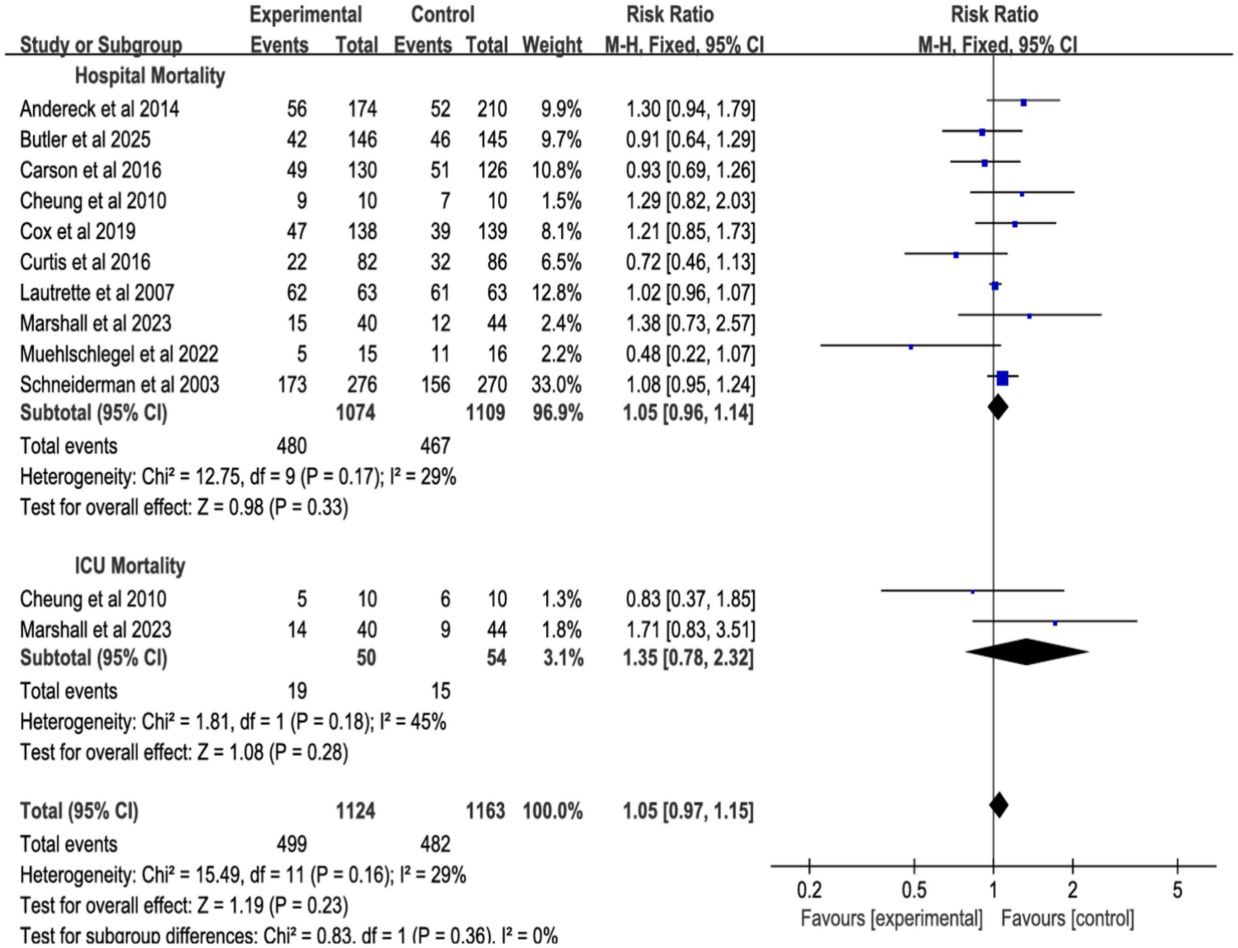
Forest plot of the effect of all-cause mortality in different environments.

The meta-analysis, incorporating 10 studies (Figure 4; Supplementary Figure S1), 10 studies showed no significant effect of shared decision-making on hospital mortality (RR = 1.05, 95% CI = 0.96–1.14, *p* = 0.33, *I*^2^ = 29%), 2 studies showed no significant effect of ICU mortality (RR = 1.35, 95% CI = 0.78–2.23, *p* = 0.28, *I*^2^ = 45%), 10 studies showed no significant effect of or overall mortality (RR = 1.05, 95% CI = 0.97–1.15, *p* = 0.23, *I*^2^ = 0%). Heterogeneity was low (<50%), and a fixed-effect model was used.

#### 3.4.2 Length of stay

**Figure 5:**
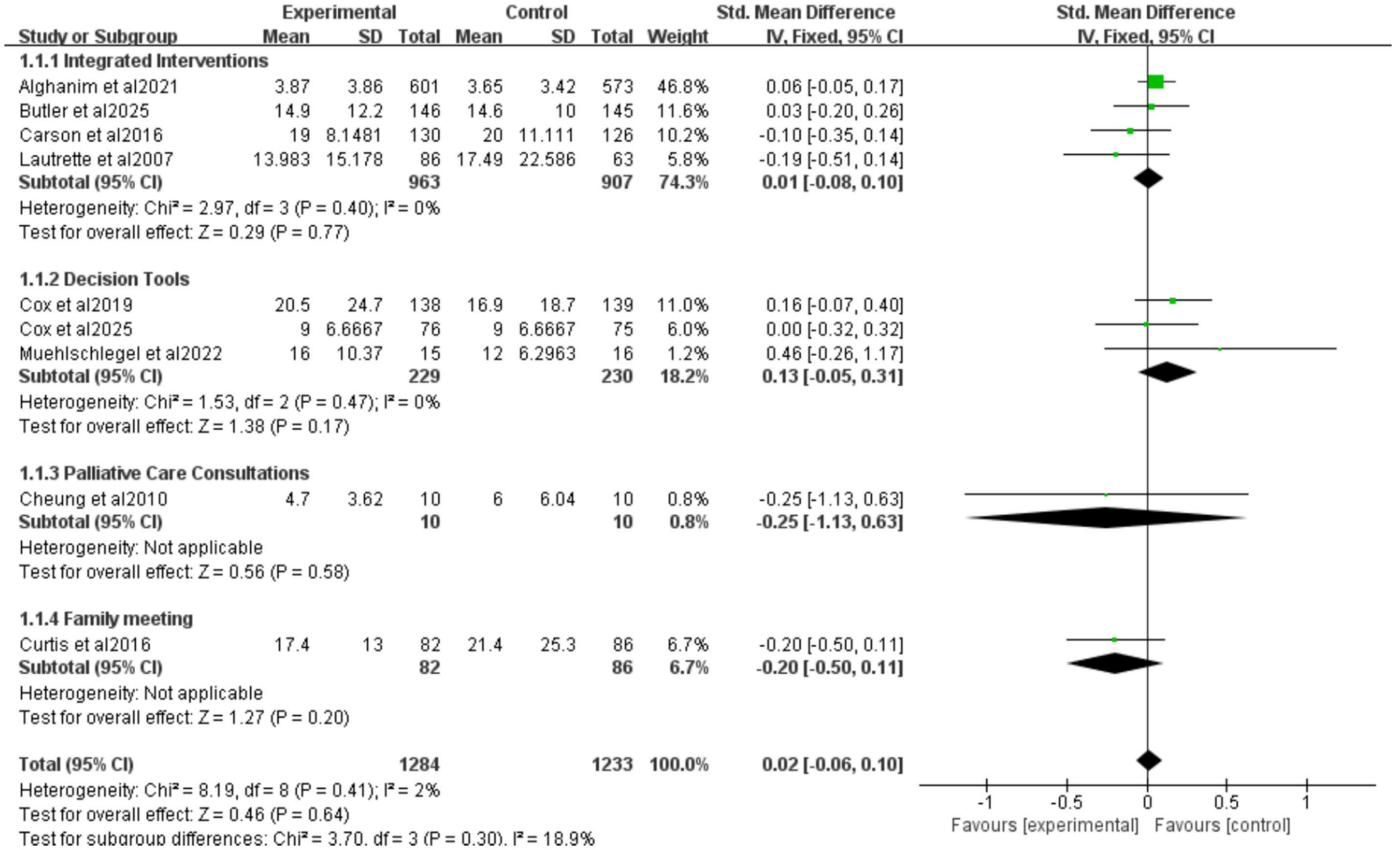
Forest plot of the effect of ICU LOS of all patients.

**Figure 6:**
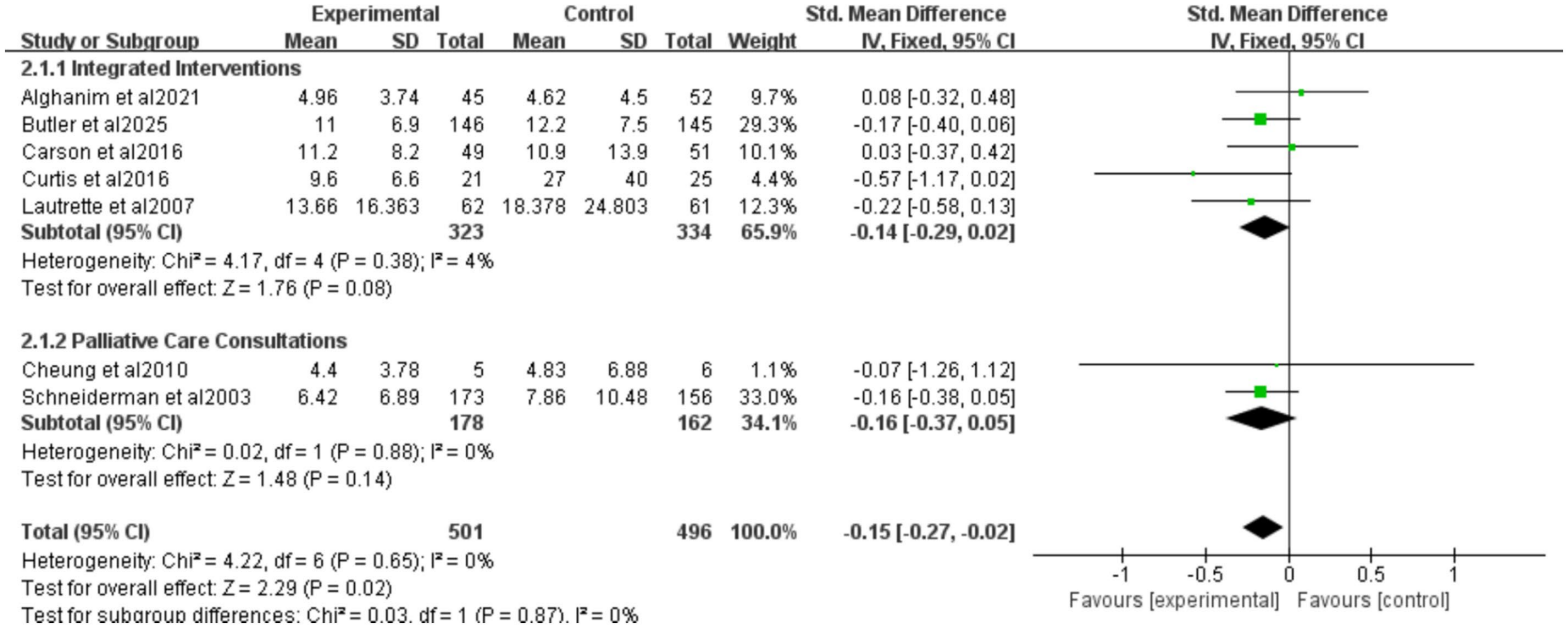
Forest plot of the effect of ICU LOS of patients who died.

**Figure 7:**
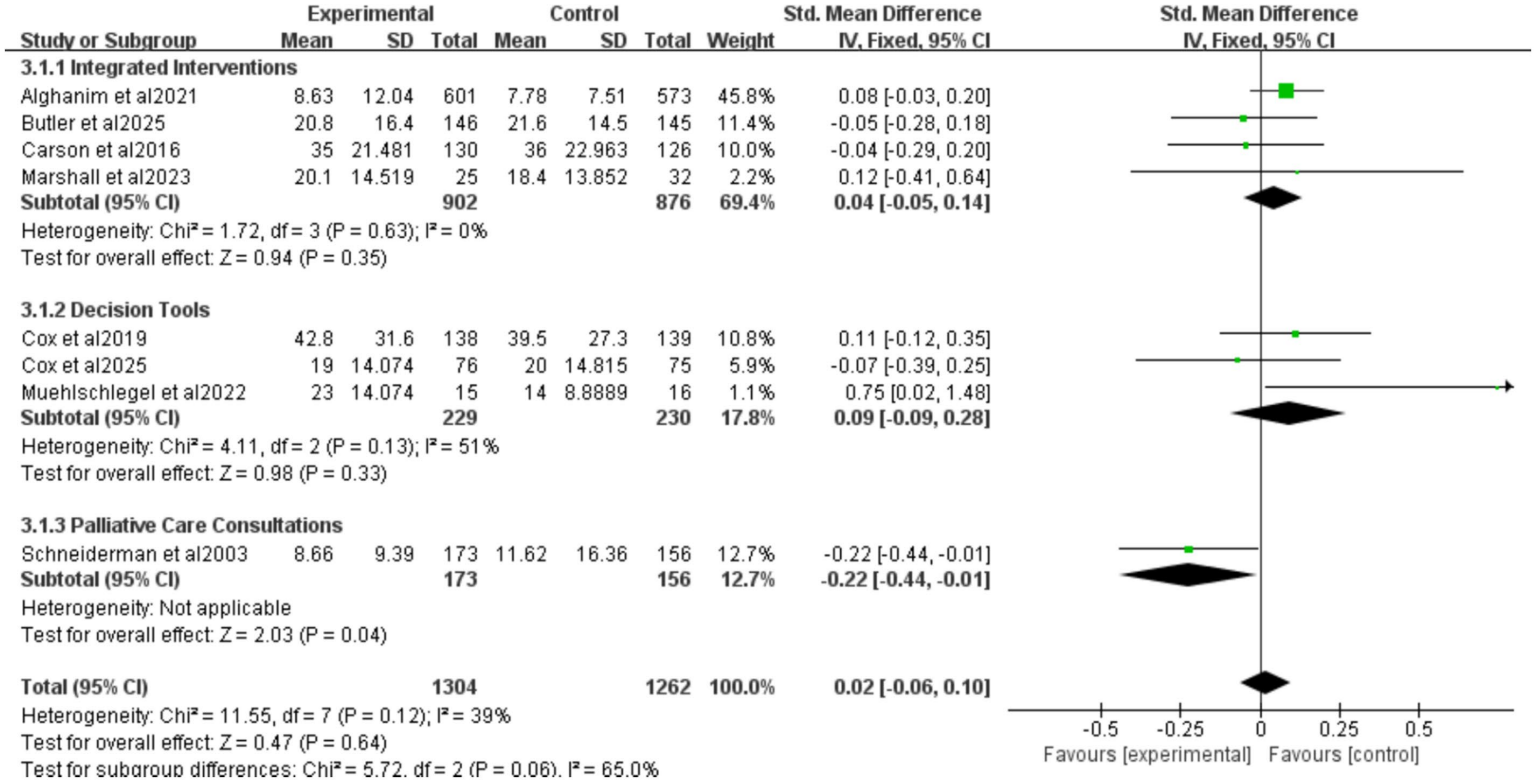
Forest plot of the effect of ICU LOS of hospital length of study.

Nine studies reported ICU length of stay (LOS) for all patients (Figure 5), seven for patients who died (Figure 6), and eight for hospital LOS (Figure 7). Shared decision-making did not reduce ICU LOS for all patients (SMD = 0.02, 95% CI = −0.06 to 0.10, *p* = 0.64, *I*^2^ = 18.9%) or hospital LOS (SMD = 0.02, 95% CI = −0.06 to 0.10, p = 0.64, *I*^2^ = 65%). However, for patients who ultimately died, shared decision-making was associated with a statistically significant reduction in ICU LOS (SMD = −0.15, 95% CI = −0.27 to −0.02, *p* = 0.02, *I*^2^ = 0%). Indicating that the relative impact of shared decision-making on this outcome is minimal and may not have practical clinical significance. Excluding any one of the studies has little influence on the total result, indicating good stability of the findings. No publication bias was detected in all the results as assessed by Egger’s test, suggesting that the pooled results are reliable (Supplementary Figures S2, S4).

##### 3.4.2.1 Intervention categorization of ICU LOS for all patients

Four studies reported integrated interventions of ICU LOS for all patients (SMD = 0.01, 95% CI = −0.08 to 0.10, *p* = 0.77, *I*^2^ = 0%), three studies reported decision tools of ICU LOS for all patients (SMD = 0.13, 95% CI = −0.05 to 0.31, *p* = 0.17, *I*^2^ = 0%), one study reported palliative care consultations of ICU LOS for all patients (SMD = −0.25, 95% CI = −1.13 to 0.63, *p* = 0.58), and one study reported family meeting of ICU LOS for all patients (SMD = −0.20, 95% CI = −0.50 to 0.11, *p* = 0.20) (Figure 5). The results were all without statistical significance. This indicates that, despite the different forms of intervention, the directional effect of shared decision-making on the length of stay in the ICU for all patients is consistent.

##### 3.4.2.2 Intervention categorization of ICU LOS for patients who died

Five studies reported integrated interventions of ICU LOS for patients who died (SMD = −0.14, 95% CI = −0.29 to 0.02, *p* = 0.08, *I*^2^ = 4%), two studies reported palliative care consultations (SMD = −0.16, 95% CI = −0.37 to 0.05, *p* = 0.14, *I*^2^ = 0%) (Figure 6). Neither subgroup was statistically significant. The “decision tools” and “family meeting” subgroups were not included in the analysis, because among the original studies included in this research, studies adopting these two intervention measures did not report data on ICU LOS for deceased patients, resulting in a lack of valid data for analysis.

##### 3.4.2.3 Intervention categorization for hospital LOS

Four studies reported integrated interventions of ICU LOS for hospital LOS (SMD = 0.04, 95% CI = −0.05 to 0.14, *p* = 0.35, *I*^2^ = 0%), three studies reported decision tools of ICU LOS for hospital LOS (SMD = 0.09, 95% CI = −0.09 to 0.28, *p* = 0.33, *I*^2^ = 51%), one study reported palliative care consultations of ICU LOS for hospital LOS (SMD = −0.22, 95% CI = −0.44 to 0.01, *p* = 0.04) (Figure 7). The “integrated intervention” and “palliative care consultation” subgroups were not included because the original studies adopting these two types of interventions did not report data on surrogate anxiety scores, resulting in a lack of valid data for analysis.

#### 3.4.3 Surrogates’ symptoms

**Figure 8:**
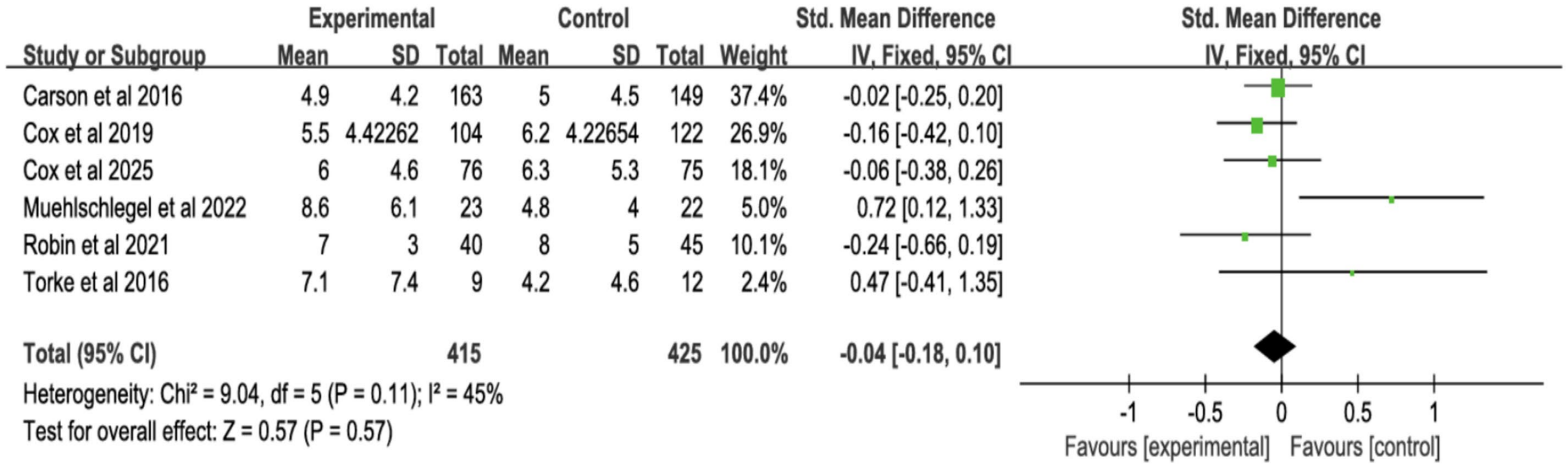
Forest plot of depression symptoms.

**Figure 9:**
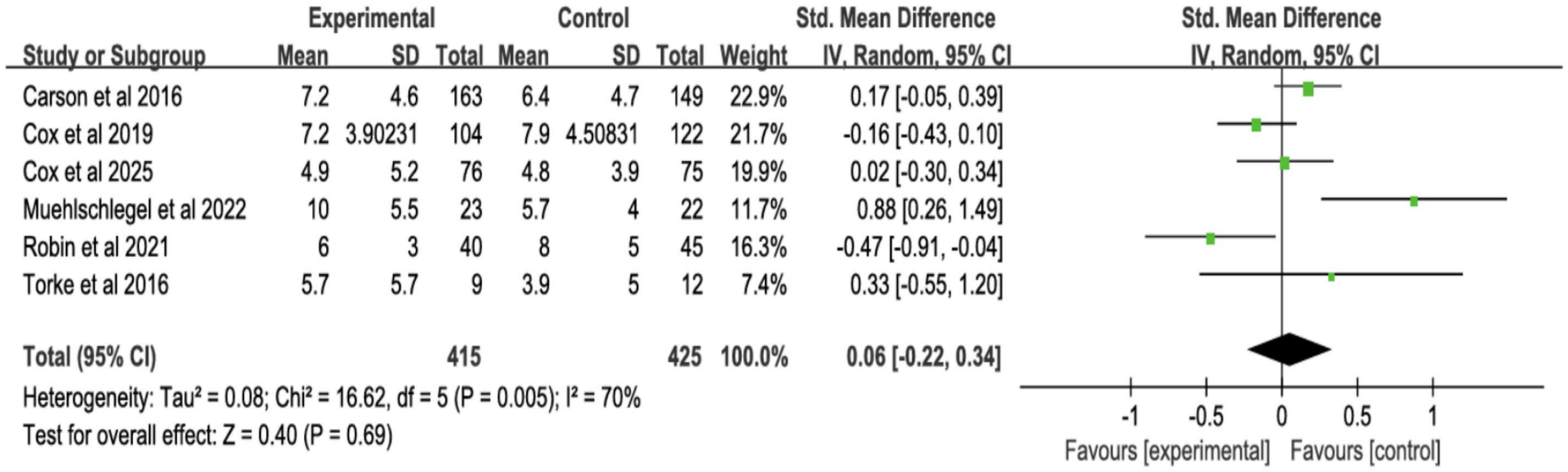
Forest plot of anxiety symptoms.

**Figure 10:**
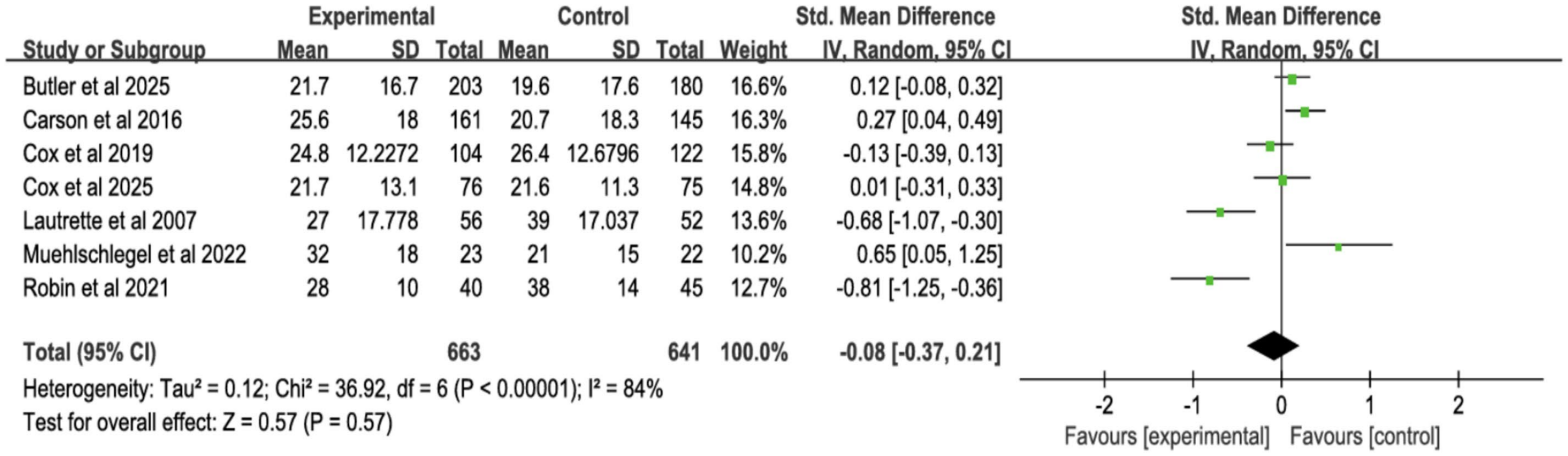
Forest plot of posttraumatic stress disorder.

Six studies assessed surrogate depression (Figure 8), six assessed anxiety (Figure 9), and seven assessed PTSD (Figure 10). Shared decision-making had no significant impact on surrogate depression (SMD = −0.04, 95% CI = −0.18 to 0.10, *p* = 0.57, *I*^2^ = 45%), anxiety (SMD = 0.06, 95% CI = −0.22 to 0.34, *p* = 0.69, *I*^2^ = 70%), or PTSD symptoms (SMD = −0.08, 95% CI = −0.37 to 0.21, *p* = 0.57, *I*^2^ = 84%). Sensitivity analyses confirmed the stability of these findings. No publication bias was detected by Egger’s test (all *p* > 0.05), indicating that the results of this study are true and reliable (Supplementary Figures S5, S7).

#### 3.4.4 Quality of decision-making and communication

**Figure 11:**
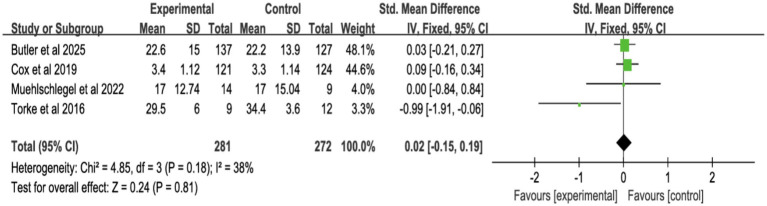
Forest plot of quality of decision-making by surrogates.

**Figure 12:**
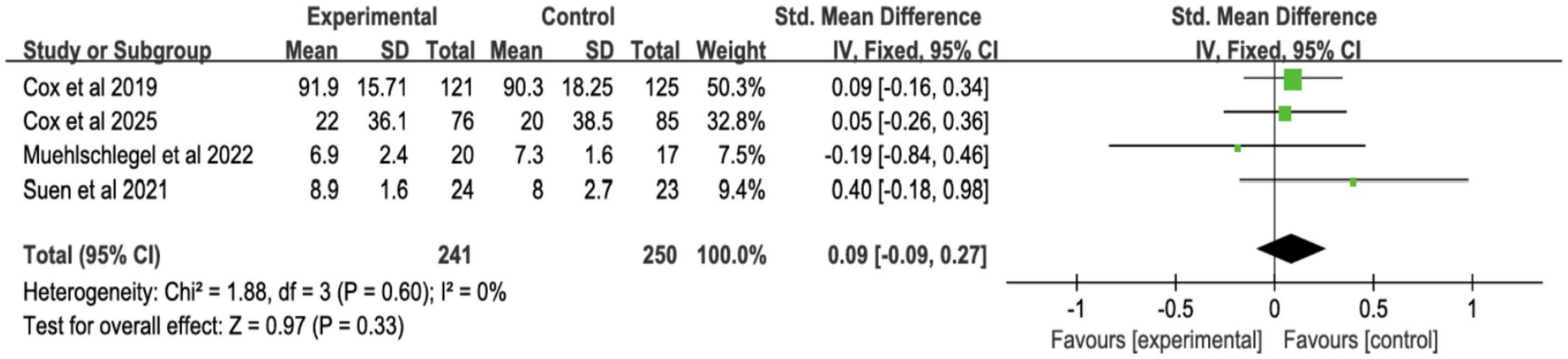
Forest plot of overall quality of communication.

Four studies reported surrogate decision-making quality (Figure 11; Supplementary Figure S8), and four reported communication quality (Figure 12; Supplementary Figure S9). Shared decision-making had no significant effect on decision-making quality (SMD = 0.02, 95% CI = −0.15 to 0.19, *p* = 0.81, *I*^2^ = 38%) or communication quality (SMD = 0.09, 95% CI = −0.09 to 0.27, *p* = 0.33, *I*^2^ = 0%).

## 4 Discussion

This review assessed the impact of shared decision-making interventions on 3,678 critically ill patients and 2,777 surrogate decision-makers across 15 RCTs. The findings indicate that shared decision-making reduced ICU LOS for deceased patients compared to standard care, consistent with previous research (33). However, shared decision-making did not significantly affect patient mortality, decision quality, or communication effectiveness, likely due to difficulties in translating shared decision-making principles into measurable clinical outcomes in the high-pressure ICU environment. By incorporating a substantial number of RCTs and larger sample sizes, this analysis strengthens the evidence base for shared decision-making’s role in improving outcomes for ICU patients and their families.

The study indicates that shared decision-making has no impact on in-hospital, ICU-specific, or all-cause mortality. A previous meta-analysis examining surrogate decision-making in critical care also found no significant effect of shared decision-making on hospital mortality rates (9). Several factors contribute to this result. ICU physicians’ prognostic inaccuracies may lead to premature withdrawal of life-sustaining treatments for patients who could recover (34). Additionally, while shared decision-making promotes collaborative decision-making, it does not fundamentally change medical protocols or treatment approaches. It mainly integrates surrogate input into existing frameworks, limiting its ability to alter care plans or mortality outcomes (35). Mortality in critically ill patients is primarily influenced by disease progression, treatment efficacy, and resource availability (36), factors largely independent of shared decision-making. Variability in intervention duration, frequency, and delivery across studies may further reduce its impact on mortality. For example, nurse-led tele-ICU support systems have been more successful in reducing ICU mortality and LOS than remote specialist consultations (10), suggesting the need for further RCTs to explore the best shared decision-making delivery models.

Our analysis reveals that shared decision-making is associated with a reduction in ICU LOS for deceased patients (SMD = −0.15), with no significant effect on overall hospital LOS. Indicating that overall shared decision-making has little or no practical impact on all these outcomes, which is contrary to the traditional common belief. A systematic review of ICU decision-making strategies also supports shared decision-making’s potential to shorten LOS specifically for non-survivors (33). However, this small difference may have limited clinical and economic significance. Regular family meetings, ethics consultations, and palliative care integration appear to improve surrogate understanding of prognosis, facilitating earlier consensus on end-of-life decisions. For patients who die, shared decision-making may prompt timely withdrawal of futile interventions, reducing unnecessary resource use. However, for surviving patients, shared decision-making’s primary value lies in enhancing collaborative decision-making rather than directly accelerating discharge. The unpredictable clinical course of critically ill patients (37) and surrogates’ varied understanding of shared decision-making, shaped by education, communication quality, and emotional stress, introduce additional complexities into LOS outcomes (38). ICU workload pressures (39), time-sensitive decisions, and surrogates’ cognitive overload may further compromise decision quality, leading to suboptimal choices (40). The importance of this finding may lie not in the importance of the effect but in its direction and consistency, it suggests that the shared decision-making process may help avoid non-beneficial life-sustaining treatments in appropriate situations, thereby achieving more efficient and patient-aligned resource utilization at the end of life. These highlight the need for shared decision-making models tailored to the stressors present in ICU settings.

As a patient-centered communication model, shared decision-making is underutilized in mental health contexts (41). A meta-analysis of shared decision-making in breast reconstruction surgery found no significant impact on anxiety levels (42), and decision aids showed limited long-term psychological benefits (11). This study confirms shared decision-making’s limited effect on surrogate mental health outcomes, including depression, anxiety, or PTSD. Surrogates’ emotional burden from poor prognoses, inconsistent clinician communication, and the psychological weight of decision-making responsibility contribute to these outcomes (43, 44). Despite these limitations, shared decision-making remains central to patient-centered care by aligning treatment plans with patient values (45). Personalized, technology-enhanced interventions and trust-building efforts within healthcare teams could optimize shared decision-making’s implementation (4), though these approaches may introduce new operational challenges for providers.

Current evidence suggests shared decision-making does not consistently improve surrogate understanding of medical decisions or enhance communication quality (46), a conclusion supported by this analysis. The complexity of shared decision-making reflects broader systemic factors: institutional culture, resource allocation, and leadership priorities shape its effectiveness (47). Clinicians often lack training in empathy and trust-building, focusing on technical discussions rather than exploring patient values (4). Heavy workloads and the lack of standardized decision aids hinder the in-depth communication needed for effective shared decision-making (5). While palliative care integration in critical care nephrology has shown benefits in decision-making quality (48), inconsistent implementation practices continue to undermine shared decision-making’s potential. Standardized protocols and rigorous outcome measures are needed to resolve ongoing debates about shared decision-making’s role in healthcare communication.

This study exclusively includes RCTs, offering robust evidence that shared decision-making reduces ICU LOS for deceased patients. However, this review has several limitations: (1) The included studies exhibited heterogeneity in the format and delivery of SDM interventions. We proactively addressed this by categorizing interventions and conducting subgroup and sensitivity analyses. These analyses indicated that while operational methods varied, the core SDM components across different intervention types yielded directionally consistent effects on primary outcomes, supporting the rationale for our pooled analysis to estimate the overall effect of the SDM process. Nevertheless, this heterogeneity underscores the need for future trials to provide more detailed descriptions of intervention components to facilitate the identification of the most effective active ingredients; (2) Excluding crossover-design RCTs may limit the comprehensiveness of outcomes due to simplified inclusion criteria; (3) The predominance of U. S.-based trials (11/15 studies) restricts generalizability to regions with distinct decision-making norms, such as Asia or Africa; (4) Including only English-language publications risks overlooking studies in other languages, which may affect the universality of our findings.

## 5 Conclusion

This systematic review suggests that implementing shared decision-making in critically ill patients may be associated with a minimal reduction in ICU length of stay for those who die, but it does not significantly improve all-cause mortality, surrogate mental health, or the quality of decision-making and communication. This finding challenges the common perception that shared decision-making yields broad benefits. Culturally adapted protocols and standardized implementation frameworks are essential to optimize shared decision-making’s role in high-stakes critical care.

## Footnotes

1
https://www.crd.york.ac.uk/PROSPERO/display_record.php?RecordID=5013215

## References

[1] Chen M Sepucha K Bozic KJ Jayakumar P. Value-based healthcare: integrating shared decision-making into clinical practice. Clin Orthop Relat Res. (2023) 481:448–50. doi: 10.1097/CORR.0000000000002580, 36735904 PMC9928684

[2] Kawamoto E Ito-Masui A Esumi R Imai H Shimaoka M. How ICU patient severity affects communicative interactions between healthcare professionals: a study utilizing wearable sociometric badges. Front Med Lausanne. (2020) 7:606987. doi: 10.3389/fmed.2020.606987, 33344484 PMC7744931

[3] Goostrey K Muehlschlegel S. Prognostication and shared decision making in neurocritical care. BMJ. (2022) 377:e060154. doi: 10.1136/bmj-2021-060154, 35696329

[4] Stalnikowicz R Brezis M. Meaningful shared decision-making: complex process demanding cognitive and emotional skills. J Eval Clin Pract. (2020) 26:431–8. doi: 10.1111/jep.13349, 31989727

[5] Baik D Cho H Masterson Creber RM. Examining interventions designed to support shared decision making and subsequent patient outcomes in palliative care: a systematic review of the literature. Am J Hospice Palliat Care. (2019) 36:76–88. doi: 10.1177/1049909118783688, 29925244 PMC6056336

[6] Vidal E I de O Kovacs M J Silva J J da Silva L. M. d. Sacardo D. P. Bersani A. L. d. F. . Position statement of ANCP and SBGG on shared decision-making in palliative care Cad Saude Publica 2022 38:e00130022. doi: 10.1590/0102-311xen130022, 36169516

[7] Wendlandt B Olm-Shipman C Ceppe A Hough CL White DB Cox CE . Surrogates of patients with severe acute brain injury experience persistent anxiety and depression over the 6 months after ICU admission. J Pain Symptom Manag. (2022) 63:e633–9. doi: 10.1016/j.jpainsymman.2022.02.336, 35595376 PMC9179180

[8] Wendlandt B Ceppe A Cox CE Hanson LC Nelson JE Carson SS. The association between patient health status and surrogate decision maker post-traumatic stress disorder symptoms in chronic critical illness. Ann Am Thorac Soc. (2021) 18:1868–75. doi: 10.1513/AnnalsATS.202010-1300OC 33794122 PMC8641832

[9] Bibas L Peretz-Larochelle M Adhikari NK Goldfarb MJ Luk A Englesakis M . Association of surrogate decision-making interventions for critically ill adults with patient, family, and resource use outcomes: a systematic review and meta-analysis. JAMA Netw Open. (2019) 2:e197229. doi: 10.1001/jamanetworkopen.2019.7229, 31322688 PMC6646989

[10] Kalvelage C Rademacher S Dohmen S Marx G Benstoem C. Decision-making authority during tele-ICU care reduces mortality and length of stay-a systematic review and meta-analysis. Crit Care Med. (2021) 49:1169–81. doi: 10.1097/CCM.0000000000004943, 33710032

[11] Xing Y Cai W Wang A Yuan Y Zhang R. Effectiveness of decision aids on critically ill patients’ outcomes and family members’ knowledge, anxiety, depression and decisional conflict: a systematic review and meta-analysis. Nurs Crit Care. (2024) 29:1303–15. doi: 10.1111/nicc.13115, 38960705

[12] Cochrane handbook for systematic reviews of interventions. (2025). Available online at: https://training.cochrane.org/handbook/current. (Accessed April, 2025)

[13] Hutton B Salanti G Caldwell DM Chaimani A Schmid CH Cameron C . The PRISMA extension statement for reporting of systematic reviews incorporating network meta-analyses of health care interventions: checklist and explanations. Ann Intern Med. (2015) 162:777–84. doi: 10.7326/M14-2385, 26030634

[14] Schneiderman LJ Gilmer T Teetzel HD Dugan DO Blustein J Cranford R . Effect of ethics consultations on nonbeneficial life-sustaining treatments in the intensive care setting: a randomized controlled trial. JAMA. (2003) 290:1166. doi: 10.1001/jama.290.9.1166 12952998

[15] Andereck WS McGaughey JW Schneiderman LJ Jonsen AR. Seeking to reduce nonbeneficial treatment in the ICU: an exploratory trial of proactive ethics intervention. Crit Care Med. (2014) 42:824–30. doi: 10.1097/CCM.0000000000000034 24201177

[16] Lautrette A Joly LM Barnoud D Joly LM Chevret S Adrie C . A communication strategy and brochure for relatives of patients dying in the ICU. N Engl J Med. (2007) 356:469–78. doi: 10.1056/NEJMoa063446 17267907

[17] Higgins JPT Altman DG Gøtzsche PC Higgins JP Jüni P Moher D . The cochrane collaboration’s tool for assessing risk of bias in randomised trials. BMJ. (2011) 343:d5928. doi: 10.1136/bmj.d5928, 22008217 PMC3196245

[18] McGrath S Katzenschlager S Zimmer AJ Seitel A Steele R Benedetti A. Standard error estimation in meta-analysis of studies reporting medians. Stat Methods Med Res. (2023) 32:373–88. doi: 10.1177/09622802221139233, 36412105

[19] McGrath S Zhao X Steele R Thombs BD Benedetti A DEPRESsion Screening Data (DEPRESSD) Collaboration. Estimating the sample mean and standard deviation from commonly reported quantiles in meta-analysis. Stat Methods Med Res. (2020) 29:2520–37. doi: 10.1177/0962280219889080, 32292115 PMC7390706

[20] Guyatt G Oxman AD Akl EA Kunz R Vist G Brozek J . GRADE guidelines: 1. Introduction-GRADE evidence profiles and summary of findings tables. J Clin Epidemiol. (2011) 64:383–94. doi: 10.1016/j.jclinepi.2010.04.026, 21195583

[21] Carson S S Cox C E Wallenstein S Hanson LC Danis M Tulsky JA et al. Effect of palliative care-led meetings for families of patients with chronic critical illness: a randomized clinical trial. JAMA, 2016, 316: 51–62. doi: 10.1001/jama.2016.8474 27380343 PMC5538801

[22] Cox CE Ashana DC Dempsey K Olsen MK Parish A Casarett D . Mobile app-facilitated collaborative palliative care intervention for critically ill older adults: a randomized clinical trial. JAMA Intern Med. (2025) 185:173–83. doi: 10.1001/jamainternmed.2024.6838, 39680398 PMC11791708

[23] Butler RA Seaman JB Felman K Stonehouse W San Pedro R Morse JQ . Randomized clinical trial of the four supports intervention for surrogate decision-makers in intensive care units. Am J Respir Crit Care Med. (2025) 211:370–80. doi: 10.1164/rccm.202405-0931OC, 39586017 PMC11936126

[24] Muehlschlegel S Goostrey K Flahive J Zhang Q Pach JJ Hwang DY. Pilot randomized clinical trial of a goals-of-care decision aid for surrogates of patients with severe acute brain injury. Neurology. (2022) 99:e1446–55. doi: 10.1212/WNL.0000000000200937, 35853748 PMC9576301

[25] Suen AO Butler RA Arnold RM Myers B Witteman HO Cox CE . A pilot randomized trial of an interactive web-based tool to support surrogate decision makers in the intensive care unit. Ann Am Thorac Soc. (2021) 18:1191–201. doi: 10.1513/AnnalsATS.202006-585OC, 33326348 PMC8328375

[26] Alghanim F Furqan M Prichett L Landon J Tao X Selvam P . The effect of chaplain patient navigators and multidisciplinary family meetings on patient outcomes in the ICU: the critical care collaboration and communication project. Crit Care Explor. (2021) 3:e0574. doi: 10.1097/CCE.0000000000000574, 34765982 PMC8577665

[27] Cox CE White DB Hough CL Jones DM Kahn JM Olsen MK . Effects of a personalized web-based decision aid for surrogate decision makers of patients with prolonged mechanical ventilation: a randomized clinical trial. Ann Intern Med. (2019) 170:285–97. doi: 10.7326/M18-2335, 30690645 PMC7363113

[28] Torke AM Wocial LD Johns SA Sachs GA Callahan CM Bosslet GT . The family navigator: a pilot intervention to support intensive care unit family surrogates. Am J Crit Care. (2016) 25:498–507. doi: 10.4037/ajcc2016730, 27802950 PMC5117831

[29] Curtis JR Treece PD Nielsen EL Gold J Ciechanowski PS Shannon SE . Randomized trial of communication facilitators to reduce family distress and intensity of end-of-life care. Am J Respir Crit Care Med. (2016) 193:154–62. doi: 10.1164/rccm.201505-0900OC, 26378963 PMC4731711

[30] Cheung W Aggarwal G Fugaccia E Thanakrishnan G Milliss D Anderson R . Palliative care teams in the intensive care unit: a randomised, controlled, feasibility study. Crit Care Resusc. (2010) 12:28–35. doi: 10.1016/S1441-2772(23)01353-4 20196711

[31] Marshall AP Van Scoy LJ Chaboyer W Chew M Davidson J Day AG . A randomised controlled trial of a nutrition and a decision support intervention to enable partnerships with families of critically ill patients. J Clin Nurs. (2023) 32:6723–42. doi: 10.1111/jocn.16752, 37161555

[32] Robin S Labarriere C Sechaud G Dessertaine G Bosson JL Payen JF. Information pamphlet given to relatives during the end-of-life decision in the ICU: an assessor-blinded, randomized controlled trial. Chest. (2021) 159:2301–8. doi: 10.1016/j.chest.2021.01.072, 33549600

[33] Kerckhoffs MC Kant M van Delden JJM Hooft L Kesecioglu J van Dijk D. Selecting and evaluating decision-making strategies in the intensive care unit: a systematic review. J Crit Care. (2019) 51:39–45. doi: 10.1016/j.jcrc.2019.01.029, 30738286

[34] Detsky ME Harhay MO Bayard DF Delman AM Buehler AE Kent SA . Discriminative accuracy of physician and nurse predictions for survival and functional outcomes 6 months after an ICU admission. JAMA. (2017) 317:2187–95. doi: 10.1001/jama.2017.4078, 28528347 PMC5710341

[35] Maley JH Wanis KN Young JG Celi LA. Mortality prediction models, causal effects, and end-of-life decision making in the intensive care unit. BMJ Health Care Inform. (2020) 27:e100220. doi: 10.1136/bmjhci-2020-100220, 33106330 PMC7592248

[36] Sala-Trull MC Monedero P Guillen-Grima F Leon-Sanz P. Mortality predictors for ICU end-of-life decisions: delta-SOFA and SAPS 3 - retrospective evaluation. BMJ Support Palliat Care. (2025) 15:spcare-2024-5357. doi: 10.1136/spcare-2024-005357, 39961643

[37] Awad A Bader-El-Den M McNicholas J Briggs J. Early hospital mortality prediction of intensive care unit patients using an ensemble learning approach. Int J Med Inform. (2017) 108:185–95. doi: 10.1016/j.ijmedinf.2017.10.002, 29132626

[38] Ke Y Cheng I Tan GSH Fok RWY Chan JJ Loh KW . Development and pilot testing of a decision aid for navigating breast cancer survivorship care. BMC Med Inform Decis Mak. (2022) 22:330. doi: 10.1186/s12911-022-02056-5, 36522635 PMC9753367

[39] Halpern SD. ICU capacity strain and the quality and allocation of critical care. Curr Opin Crit Care. (2011) 17:648–57. doi: 10.1097/MCC.0b013e32834c7a53, 21986461

[40] Hua M Halpern SD Gabler NB Wunsch H. Effect of ICU strain on timing of limitations in life-sustaining therapy and on death. Intensive Care Med. (2016) 42:987–94. doi: 10.1007/s00134-016-4240-8, 26862018 PMC4846491

[41] Chmielowska M Zisman-Ilani Y Saunders R Pilling S. Trends, challenges, and priorities for shared decision making in mental health: the first umbrella review. Int J Soc Psychiatry. (2023) 69:823–40. doi: 10.1177/00207640221140291, 36680367 PMC10240653

[42] Chen L Lu J Chen B Zhang X. Effect of shared decision-making in patients with breast cancer undergoing breast reconstruction surgery: a systematic review and meta-analysis. Asia Pac J Oncol Nurs. (2024) 11:100596. doi: 10.1016/j.apjon.2024.100596, 39582550 PMC11582372

[43] Fattori F Zisman-Ilani Y Chmielowska M Rodríguez-Martín B. Measures of shared decision making for people with mental disorders and limited decisional capacity: a systematic review. Psychiatr Serv. (2023) 74:1171–5. doi: 10.1176/appi.ps.202200018, 37194313

[44] Hempeler C Gather J Haberstroh J Trachsel M. Shared decision-making for patients with mental disorders or cognitive impairments. Therapeut Umschau Rev Therapeut. (2022) 79:393–400. doi: 10.1024/0040-5930/a001380, 36164732

[45] Coulter A. Shared decision making: everyone wants it, so why isn’t it happening? World Psychiatry. (2017) 16:117–8. doi: 10.1002/wps.20407, 28498596 PMC5428189

[46] Vick JB Berger BT Ubel PA Cox CE You HB Ma JE . Shared decision-making communication and prognostic misunderstanding in the ICU. JAMA Netw Open. (2024) 7:e2439715. doi: 10.1001/jamanetworkopen.2024.39715, 39405057 PMC11581528

[47] Scholl I LaRussa A Hahlweg P Kobrin S Elwyn G. Organizational- and system-level characteristics that influence implementation of shared decision-making and strategies to address them - a scoping review. Implement Sci. (2018) 13:40. doi: 10.1186/s13012-018-0731-z, 29523167 PMC5845212

[48] Hemmat V Corbett C. Palliative care for nephrology patients in the intensive care unit. Crit Care Nurs Clin North Am. (2022) 34:467–79. doi: 10.1016/j.cnc.2022.07.003, 36336436

