# Supplementary material for: The effect of shared decision-making for critically ill patients: a systematic review and meta-analysis
Source: Front Med (Lausanne). 2026 Jan 30;13:1726976. doi: 10.3389/fmed.2026.1726976 (PMC12901507; doi:10.3389/fmed.2026.1726976)
Supplement: Supplementary file 1 [file Table_1.docx]

Table S1. Search strategy of PubMed

PubMed （Date of search2025.3.18）

| Query | Hits |
| --- | --- |
| #1 Decision Making, Shared[MeSH Terms] | 2689 |
| #2 Decision making[MeSH Terms] | 246290 |
| #3 Decision Support Techniques[MeSH Terms] | 84384 |
| #4((((((Decision Making, Shared[Title/Abstract]) OR (Decision making[Title/Abstract])) OR (Decision Support[Title/Abstract])) OR (shared decision making[Title/Abstract])) OR (decision aid[Title/Abstract])) OR (Palliative Car*[Title/Abstract])) OR (decision[Title/Abstract]) | 479696 |
| #5 #1 or #2 or #3 or #4 | 721545 |
| #6 Critical Care Nursing[MeSH Terms] | 3013 |
| #7 Critical Care[MeSH Terms] | 69574 |
| #8 Intensive Care Units[MeSH Terms] | 115311 |
| #9 Critical illness[MeSH Terms] | 42553 |
| #10 (((Critical Care Nursing[Title/Abstract]) OR (Critical Care[Title/Abstract])) OR (Intensive Care Units[Title/Abstract])) OR (Critical Illness[Title/Abstract]) | 95309 |
| #11 #6 or #7 or #8 or #9 or #10 | 235339 |
| #12 Family[MeSH Terms] | 388283 |
| #13 Caregivers[MeSH Terms] | 56664 |
| #14 Proxy[MeSH Terms] | 1887 |
| #15(((((famil*[Title/Abstract]) OR (carer[Title/Abstract])) OR (Caregiver[Title/Abstract])) OR (prox*[Title/Abstract])) OR (surrogate decision maker*[Title/Abstract])) OR (decision maker*[Title/Abstract]) | 1852713 |
| #16 #12 or #13 or #14 or #15 | 2107002 |
| #17 #5 and #11 and #16 | 4182 |

Table S2. Search strategy of Web of Science

Web of Science （Date of search2025.3.18）

| Query | Hits |
| --- | --- |
| 1 (((((((((TS=( Decision Making, Shared)) OR TS=(Decision making)) OR TS=(Decision Support Techniques)) OR AB=(Decision Making, Shared)) OR AB=(Decision making)) OR AB=(Decision Support)) OR AB=(shared decision making)) OR AB=(decision aid)) OR AB=(Palliative Car*)) OR AB=(decision) | 2384049 |
| 2 (((((((TS=(Critical Care Nursing)) OR TS=(Critical Care)) OR TS=(Intensive Care Units)) OR TS=(Critical illness)) OR AB=(Critical Care Nursing)) OR AB=(Critical Care)) OR AB=(Intensive Care Units)) OR AB=(Critical Illness) | 640879 |
| 3 ((((((((TS=(Family)) OR TS=(Caregivers)) OR TS=(Proxy)) OR AB=(famil*)) OR AB=(carer)) OR AB=(Caregiver)) OR AB=(prox*)) OR AB=(surrogate decision maker*)) OR AB=(decision maker*) | 4490206 |
| 4 #1 AND #2 AND #3 | 14334 |

Table S3. Search strategy of Embase

Embase （Date of search2025.3.18）

| Query | Hits |
| --- | --- |
| 1'shared decision making'/exp | 19787 |
| 2'decision making'/exp | 515661 |
| 3'decision support system'/exp | 40025 |
| 4'decision making, shared':ab,ti OR 'decision making':ab,ti OR 'decision support':ab,ti OR 'shared decision making':ab,ti OR 'decision aid':ab,ti OR 'palliative car*':ab,ti OR 'decision':ab,ti | 654678 |
| 5 #1 or #2 or #3 or #4 | 942580 |
| 6'critical care nursing'/exp | 3918 |
| 7 'critical care'/exp | 983177 |
| 8 'intensive care units'/exp | 347081 |
| 9 'Critical illness'/exp | 38404 |
| 10'critical care nursing':ab,ti OR 'critical care':ab,ti OR 'intensive care units':ab,ti OR 'critical illness':ab,ti | 128705 |
| 11 #6 or #7 or #8 or #9 or#10 | 1240593 |
| 12 'family'/exp | 665403 |
| 13 'caregiver'/exp | 133557 |
| 14 'proxy'/exp | 1557 |
| 15'famil*':ab,ti OR 'carer':ab,ti OR 'caregiver':ab,ti OR 'prox*':ab,ti OR 'surrogate decision maker*':ab,ti OR 'decision maker*':ab,ti | 2347549 |
| 16 #12 or #13 or #14 or #15 | 2800875 |
| 17 #5 and #11 and #16 | 12667 |

Table S4. Search strategy of Cochrane Library

Cochrane Library（Date of search2025.3.18）

| Query | Hits |
| --- | --- |
| #1 MeSH descriptor: [Decision Making, Shared] explode all trees | 218 |
| #2 MeSH descriptor: [Decision Making] explode all trees | 6553 |
| #3 MeSH descriptor: [Decision Support Techniques] explode all trees | 4512 |
| #4 (Decision Making, Shared):ti,ab,kw OR (Decision making):ti,ab,kw OR (Decision Support):ti,ab,kw AND (shared decision making):ti,ab,kw OR(decision aid):ti,ab,kw OR(Palliative Car*):ti,ab,kw OR(decision):ti,ab,kw | 44201 |
| #5 #1 or #2 or #3 or #4 | 49489 |
| #6 MeSH descriptor: [Critical Care Nursing] explode all trees | 81 |
| #7 MeSH descriptor: [Critical Care] explode all trees | 3028 |
| #8 MeSH descriptor: [Intensive Care Units] explode all trees | 6115 |
| #9 MeSH descriptor: [Critical Illness] explode all trees | 3729 |
| #10 (Critical Care Nursing):ti,ab,kw OR (Critical Care):ti,ab,kw OR (Intensive Care Units):ti,ab,kw AND (Critical Illness):ti,ab,kw | 15739 |
| #11 #6 or #7 or #8 or #9 or #10 | 21133 |
| #12 MeSH descriptor: [Family] explode all trees | 14952 |
| #13 MeSH descriptor: [Caregivers] explode all trees | 3954 |
| #14 MeSH descriptor: [Proxy] explode all trees | 88 |
| #15 (famil*):ti,ab,kw OR (carer):ti,ab,kw OR (Caregiver):ti,ab,kw AND (prox*):ti,ab,kw OR(surrogate decision maker*):ti,ab,kw OR(decision maker*):ti,ab,kw | 68951 |
| #16 #12 or #13 or #14 or #15 | 78388 |
| #17 #5 and #11 and #16 | 447 |

Table S5

| Certainty assessment | | | | | | | No of patients | | Effect | | Certainty | Importance |
| --- | --- | --- | --- | --- | --- | --- | --- | --- | --- | --- | --- | --- |
| No of studies | Study design | Risk of bias | Inconsistency | Indirectness | Imprecision | Other considerations | shared decision-making | usual care | Relative(95% CI) | I^2^ |  |  |
| All-cause mortality in different environments | | | | | | | | | | | | |
| Hospital mortality | | | | | | | | | | | | |
| 10 | RCTs | serious^a^ | not serious | not serious | not serious | none | 480/1074 (44.7%) | 467/1109 (42.1%) | RR=1.05  (0.96 to 1.14) | 29% | ⨁⨁⨁◯Moderate^a^ | CRITICAL |
| ICU mortality | | | | | | | | | | | | |
| 2 | RCTs | serious^a^ | not serious | not serious | serious^b^ | none | 19/50 (38.0%) | 15/54 (27.8%) | RR=1.35  (0.78 to 2.32) | 45% | ⨁⨁◯◯Low^a,b^ | CRITICAL |
| ICU length of stay of all patients | | | | | | | | | | | | |
| 9 | RCTs | serious^a^ | not serious | not serious | not serious | none | 1284 | 1233 | SMD=0.02  (-0.06 to 0.10) | 2% | ⨁⨁⨁◯Moderate^a^ | CRITICAL |
| ICU length of stay of patients who died | | | | | | | | | | | | |
| 7 | RCTs | serious^a^ | not serious | not serious | not serious | none | 501 | 496 | SMD=-0,15  (-0.27 to -0.02) | 0% | ⨁⨁⨁◯Moderate^a^ | CRITICAL |
| Hospital length of stay | | | | | | | | | | | | |
| 8 | RCTs | serious^a^ | not serious | not serious | not serious | none | 1304 | 1262 | SMD=0.02  (-0.06 to 0.10) | 39% | ⨁⨁⨁◯Moderate^a^ | CRITICAL |
| Depression symptoms | | | | | | | | | | | | |
| 6 | RCTs | serious^a^ | not serious | not serious | not serious | none | 415 | 425 | SMD=-0.04  (-0.18 to 0.10) | 45% | ⨁⨁⨁◯Moderate^a^ | IMPORTANT |
| Anxiety symptoms | | | | | | | | | | | | |
| 6 | RCTs | serious^a^ | serious^c^ | not serious | not serious | none | 415 | 425 | SMD=0.06  (-0.22 to 0.34) | 70% | ⨁⨁◯◯Low^a,c^ | IMPORTANT |
| Posttraumatic stress disorder | | | | | | | | | | | | |
| 7 | RCTs | serious^a^ | very serious^d^ | not serious | not serious | none | 663 | 641 | SMD=-0.08  (-0.37 to 0.21) | 84% | ⨁◯◯◯Very low^a,d^ | IMPORTANT |
| Quality of decision-making by surrogates | | | | | | | | | | | | |
| 4 | RCTs | serious^a^ | not serious | not serious | not serious | none | 281 | 272 | SMD=0.02  (-0.15 to 0.19) | 38% | ⨁⨁⨁◯Moderate^a^ | IMPORTANT |
| Overall quality of communication | | | | | | | | | | | | |
| 4 | RCTs | serious^a^ | not serious | not serious | not serious | none | 241 | 250 | SMD=0.09  (-0.09 to 0.27) | 0% | ⨁⨁⨁◯Moderate^a^ | IMPORTANT |

CI: Confidence interval; RR: risk ratio

a. due to over 1/2 from middle or high risk

b. due to sample size<300

c. due to 50%<I^2^<75%

d. due to I^2^≥75%

Table 1

| **Author, year,**  **country** | **Sites** | **Participants** | **Intervention** | **No. of participants**  **(experimental/control)** | | **Age**  **(years, experimental / control, mean ± SD)** | | **Outcome and outcome measure** |
| --- | --- | --- | --- | --- | --- | --- | --- | --- |
|  |  |  |  | **Patients(male/female)** | **SDMs(male/female)** | **Patients** | **SDMs** |  |
| Cox et al, 2025, US | Multicenter RCT of 6 ICUs | Critically ill older adult patients and their family members with elevated palliative care needs | An automated electronic health record–integrated, mobile application–based communication platform | 76(42/34)/75(44/31) | 76(22/53)/75(18/57) | 69.2±9.8/70.5±9.6 | 57.3±12.9/57.4±13.0 | Depression, anxiety, surrogates’ symptoms of posttraumatic stress disorder, ICU LOS (all patients), hospital LOS, overall QOC |
| Butler et al, 2025, US | Multicenter RCT of 6 ICUs | Patients ≥21 years old, with >40% risk of in-hospital death or long-term impairment, dependent on >2 ADLs, and their surrogates | Four Supports intervention adds: emotional support; communication support; decisional support | 146(75/71)/145(88/57) | 233（55/168）/209(63/146) | 61.0±17.1/62.2±15.6 | 51.1±15.0/53.4±15.2 | Hospital mortality, surrogates’ symptoms of posttraumatic stress disorder, ICU LOS (all patients), ICU LOS (patients who died), hospital LOS, quality of decision-making surrogate |
| Marshall et al, 2023, Australia | Multicenter RCT of 9 ICUs | ICU patients who were nutritionally high-risk and/or those at risk of dying in the ICU or during subsequent hospitalisation and their adult family members | Nutrition intervention and decision support intervention | 40(25/15)/44(31/13) | 40(12/28)/44(11/33) | 73.0 ± 8.0/71.5 ± 8.2 | 56.4 ± 16.2/58.1 ± 16.1 | Hospital mortality, ICU mortality, hospital LOS |
| Muehlschlegel et al, 2022, US | Multicenter RCT of 2 ICUs | Critically ill patients with severe acute brain injury and their surrogates | Provided a technical orientation of the decision-aid to the intervention group surrogates without any mention of medical information and reminded them to complete the worksheet | 20(11/9)/21(15/6) | 33(5/27)/33(11/22) | 58 ± 19/64 ± 21 | 51 ± 17/57 ± 15 | Hospital mortality, depression, anxiety, surrogates’ symptoms of posttraumatic stress disorder, ICU LOS (all patients), hospital LOS, quality of decision-making surrogate, overall QOC, length of mechanical ventilation |
| Suen et al, 2021, US | Single center RCT of 2 ICUs | Critical patients in ICU and their surrogates | Surrogates complete Family Support Tool before meetings, provide summary to ICU team, and schedule family meetings | 25(13/12)/25(11/14) | 23(6/17)/25(12/13) | 65.6±17.7/69±13.2 | 58.65±12.62/54.28±13.15 | Overall QOC |
| Robin et al, 2021, France | Single center RCT of 3 ICUs | Adult patients for whom a decision to withhold and withdraw life-sustaining therapies in the ICU | Information pamphlet | 45(28/17)/45(29/16) | 45(20/25)/45(14/31) | 69 (62-77)/71 (59-80) | 54 (47–65)/54 (47–66) | Depression, anxiety, surrogates’ symptoms of posttraumatic stress disorder |
| Alghanim et al, 2021, US | Single center RCT of 2 ICUs | Critical patients in MICU or CICU and their surrogates | Chaplain patient navigator served as a health liaison for patients and their families, elicited their values and preferences, and organized admission, follow-up, and ad hoc family meetings | 601(334/266)/573(287/286) | Not mentioned | 63(53-76)/62(53-75) | Not mentioned | ICU LOS (all patients), ICU LOS (patients who died), hospital LOS |
| Cox et al, 2019, US | Multicenter RCT of 5 hospital | Adult patients receiving prolonged mechanical ventilation and their surrogates | Web-based decision aid provided prognostic estimates, treatment options, and clarified patient values for family meetings. | 138(88/50)/139(89/50) | 137(41/96)/138(33/105) | 52.9 ± 17.9/54.0 ± 16.6 | 49.9 ± 13.5/52.6 ± 11.6 | Hospital mortality, depression, anxiety, surrogates’ symptoms of posttraumatic stress disorder, ICU LOS (all patients), hospital LOS, quality of decision-making surrogate, overall QOC, length of mechanical ventilation |
| Torke et al, 2016, US | Single center RCT | Sedated or comatose ICU patients | Dedicated trained nurse acting as family navigator | 13(4/9)/13(7/6) | 13(9/4)/13(3/10) | 53.27 (14.18)/ 57.42 (11.03) | 50.93 (12.01)/ 46.16 (17.36) | Depression, anxiety, quality of decision-making surrogate |
| Curtis et al, 2016, US | Multicenter RCT of 2 ICUs | ICU patients and family members | A communication facilitator enhances ICU communication self-efficacy for families and clinicians through interviews, meetings, and follow-up | 82(55/27)/86(53/33) | 131(38/93)/137(41/96) | 52.1±17.2/55.3±18.8 | 49.5±12.0/52.4±14.2 | Hospital mortality, ICU LOS (all patients), ICU LOS (patients who died) |
| Carson et al, 2016, US | Multicenter RCT of 4 ICUs | Adult patients (≥21 years) requiring 7 days of mechanical ventilation, with their family surrogates enrolled in the study | At least 2 structured family meetings led by palliative care specialists and provision of an informational brochure | 130(64/66)/120(55/65) | 184(56/128)/181(50/131) | 58 (55.2–60.8)/57 (54.0–59.7) | 51 (48.8–52.8)/51 (48.6–52.7) | Hospital mortality, depression, anxiety, surrogates’ symptoms of posttraumatic stress disorder, ICU LOS (all patients), ICU LOS (patients who died), hospital LOS, length of mechanical ventilation |
| Cheung et al, 2010, Australia | Single center RCT | Patients with terminal or preterminal conditions, where escalating or continuing treatment is unlikely to improve their clinical condition | A consultation and subsequent management by a palliative care team | 10(5/5)/10(3/7) | 共9 | 72(20)/83(14) | Total 9 | Hospital mortality, ICU mortality, ICU LOS (all patients), ICU LOS (patients who died) |
| Andereck et al, 2014, US | Single center RCT of medical/surgical ICU | Patients with ICU lengths of stay of five days or greater | Ethics: Proactive ethics intervention involves a trained bioethicist in the care of all ICU patients | 174(83/91)/210(98/112) | 146/173 | 60±15.8/61±16.2 | Not reported | Hospital mortality |
| Lautrette et al, 2007, France | Multicenter RCT of 22 ICUs | Patient would die within a few days | A proactive end-of-life conference and a brochure | 63(33/30)/63(37/26) | 57(17/40)/52(12/40) | 74(56-80)/68(56-76) | 54(47-58)/54(46-64) | Hospital mortality, surrogates’ symptoms of posttraumatic stress disorder, ICU LOS (all patients), ICU LOS (patients who died) |
| Schneiderman et al, 2003, US | Multicenter RCT of 7 hospitals | Patient in whom treatment conflicts were identified | Ethics consultation offered | 276(145/131)/270(148/122) | 262/263 | 67.5±17.2/67.5±17.4 | Not reported | Hospital mortality, ICU LOS (patients who died), hospital LOS, length of mechanical ventilation |

SDM = surrogate decision-maker, RCT = randomized controlled trial, ICU= intensive care unit, ICU LOS = intensive care unit length of study, LOS = length of study, QOC = quality of communication
